# High-risk human papilloma virus and cervical abnormalities in HIV-infected women with normal cervical cytology

**DOI:** 10.1186/1750-9378-9-36

**Published:** 2014-11-03

**Authors:** Jonah Musa, Chad Achenbach, Babafemi Taiwo, Baiba Berzins, Olugbenga Silas, Patrick H Daru, Oche Agbaji, Godwin Imade, Atiene S Sagay, John A Idoko, Phyllis J Kanki, Robert L Murphy

**Affiliations:** Department of Obstetrics and Gynecology, University of Jos, Jos, Plateau State Nigeria; Center for Global Health, Northwestern University, Chicago, IL USA; Department of Pathology, University of Jos, Jos, Plateau State Nigeria; Department of Medicine, University of Jos, Jos, Plateau State Nigeria; Department of Immunology and Infectious Diseases, Harvard School of Public Health, Boston, MA USA; AIDS Prevention Initiative in Nigeria, HIV program, Jos University Teaching Hospital, Jos, Plateau State Nigeria

**Keywords:** HR-HPV, HIV, Cervical cytology, Cervical cancer, Nigeria

## Abstract

**Background:**

The prevalence of High-Risk Human papilloma virus (HR-HPV), a necessary cause of invasive cervical cancer (ICC) is relatively high in HIV infected women. Gaps exist in our knowledge of the optimal approaches for managing women who have HR-HPV with normal cervical cytology (NCC) particularly in settings of HIV infection.

**Methods:**

Between May 2012 and June 2013 we conducted a colposcopic assessment of HIV-infected women with prior (NCC) and known HR-HPV status to compare cervical abnormalities in women with and without HR-HPV. Colposcopic examinations were done at the Operation Stop Cervical Cancer (OSCC) unit of the Jos University Teaching Hospital (JUTH), Jos, Nigeria. Abnormal colposcopic finding (ACF) was defined as areas of aceto-white epithelium involving the squamo-coulumnar junction, areas of punctation, mosaic pattern or atypical vessels. We compared proportions of ACF as well as histologic grades of cervical intra-epithelial neoplasia (CIN) in women with or without HR-HPV. Statistical analysis was done on STATA.

**Results:**

We conducted colposcopic examinations in 78 out of 89 (86.5%) eligible women. The mean age of the cohort was 32.4 years (SD ±4.6) with a median 32 years (IQR 29–36). After a mean follow up time of 20.1 months from the initial cervical pap cytology and HR-HPV testing, we found 12 of 78 (15.4%) women with ACF. The odds for an ACF was statistically higher [OR = 4.0 (95% CI: 1.1-14.7)] in women with HR-HPV compared to those without. Of the twelve women with ACF, subsequent histologic examination of colposcopically directed cervical biopsies confirmed CIN 1 in 4 cases (33.3%), CIN 2 in 1 case (8.3%), CIN 3 in 2 cases (16.7%), carcinoma-in-situ (CIS) in 2 cases (16.7%), and normal cervix in 3 (25.0%). Overall, the proportion of women detected with any grade of CIN was 11.5% (9/78) and 6.4% (5/78) were CIN 2 or greater lesion (CIN2+).

**Conclusion:**

HIV-infected women with NCC and HR-HPV had a four-fold higher likelihood for an ACF. The practice of early colposcopic examination of HIV-infected women with prior NCC and HR-HPV may increase early detection of higher grade CIN and CIS cancer stages in our setting.

## Introduction

Cervical cancer was responsible for up to 25.4% of all new cancer cases in women in sub-Saharan Africa in 2012 [1]. Nigeria has a large burden of invasive cervical cancer and mortality [2] is equally high due to late presentation and limited treatment facilities. In Nigeria, 11,431 cases of cervical cancer and 5,952 cervical cancer deaths were reported in 2010 compare to 5,714 cases and 3,158 cervical cancer deaths in 1980 in the same country [2]. The high burden of human immuno deficiency virus (HIV) among women in Nigeria contributes to the growing incidence of premalignant lesions of the cervix as well as progression to invasive cervical cancer, necessitating the integration of cervical screening efforts using visual inspection with acetic acid (VIA) into existing HIV prevention, care and treatment programs in Nigeria [3].

In developed countries of the world, well-organized cervical cancer screening programs using Pap smear cytology has led to significant declines in invasive cervical cancer. Such national programs do not exist in most developing countries like Nigeria. Studies have shown that women with HIV have an increase risk of having high grade squamous intraepithelial lesions (SILs) with higher rates of progression to invasive stages [4, 5]. Studies on the association of SILs and degree of HIV immunosupression have documented a significant risk in some and not others [3, 6, 7]. The role of high-risk human papilloma virus (HR-HPV) in cervical carcinogenesis has been well established from epidemiologic studies [8]. Studies have demonstrated the effectiveness of HR-HPV DNA testing in detecting high-grade squamous intraepithelial lesions and prevention of invasive cervical cancer cases compare to cervical cytology [9–12]. Also, the utility of HR-HPV DNA testing as a cervical cancer prevention strategy has demonstrated significant reduction in cervical cancer mortality compare to conventional Pap cytology in resource limited countries [13].

Additionally, studies have shown that even in settings where cervical cancer screening is available, conventional Pap cytology is associated with high false negative and positive results [14] and some high-grade cervical lesions have been missed in women with normal cervical cytology reports. The Athena trial [15] and a prevalence study in India have shown the potential benefits of HR-HPV testing, for the detection of high-grade cervical lesions among women with either atypical squamous intraepithelial lesions of undetermined significance or a negative pap smear cytology [16].

Gaps exist in the knowledge of optimal management approaches for women with normal pap cervical cytology and a positive HR-HPV particularly in the setting of HIV infection. To assess the possible role of HR-HPV DNA testing in detecting underlying high-grade cervical lesions in women with prior NCC in the setting of HIV infection, we conducted a pilot study of HR-HPV DNA testing in women with NCC in a cohort of HIV infected women in Jos, Nigeria. The prevalence and epidemiologic factors associated with HR-HPV in our patient population have been previously reported [17]. The current report focused on colposcopic examination findings and cervical abnormalities in women with or without HR-HPV in our cohort.

## Methods

### Study setting, study population and procedures

Between May 2012 and June 2013, we enrolled a cohort of HIV-infected women with NCC at the Reproductive Health Unit (RHU) of APIN of the APIN/Harvard PEPFAR HIV Clinic, Jos University Teaching Hospital, (JUTH) Jos, Nigeria. The RHU was set up in 2008 and is staffed by two reproductive health nurses and a gynecologist to address issues related to contraception and cervical cancer screening.

Routine care at JUTH includes CD4+ T cell (Partec, Gemany) and viral load (Roche Amplicor 1.5, lower detection limit 400 copies/ml) measurement before antiretroviral therapy (ART), then approximately every 3 months during ART. Initiation of ART follows the Nigerian National Guidelines for HIV and AIDS treatment and care in adolescents and adults, 2007 [18]. Other information routinely collected in the clinical care of patients at JUTH included the following: demographics, ART history and co-infections (hepatitis B, hepatitis C and tuberculosis). At enrollment, each patient gave written informed consent for collection and use of their medical information for research purposes.

A detailed questionnaire was administered to each participant to determine age, age at first coitus, parity, duration of HIV infection, ART history, previous abortions, history of contraception, previous Pap smear, smoking and alcohol consumption. Patient information not recalled by study participants such as duration of HIV disease, ART history etc. were extracted from their electronic database.

During the first part of this study, this cohort of women had cervical samples obtained for HR-HPV diagnosis by Hybrid Capture 2 (HC2) signal amplification (Digene Corporation, Gaithersburg, USA. The HC2 test is qualitative and a positive test indicates presence of one or more of the following 13 sub-types of HR-HPV: 16, 18, 31, 33, 35, 39, 45, 51, 52, 56, 58, 59 or 68. Other details of the enrollment setting and sampling procedures for Pap smear preparation and interpretation has been described in the preliminary published work by Musa et al. [17]. At the time of enrollment, informed consent was obtained for a follow up visit during which a colposcopic examination would be done to detect any abnormality in the cervix.

The colposcopic examinations were performed at the Operation Stop Cervical Cancer (OSCC) unit of the department of Obstetrics and Gynecology, JUTH. The study participants were contacted through phone calls by the study nurse and a study examination visit was scheduled. In situations where the participants were not successfully contacted through phone calls (“wrong numbers”, “number not available”, etc.), the researchers made efforts to track participants at their pharmacy drug pick-up visits where they received their regular antiretroviral drugs at the Adult HIV Treatment Clinic of JUTH. The colposcopic examinations were performed by a consultant gynecologist trained in VIA and colposcopic examination of the cervix. The gynecologist conducting the colposcopic examination was blinded to the HR-HPV status of the participant. Women who had detectable abnormalities were treated according to standard guidelines in our hospital. Abnormal colposcopic finding was defined as areas of aceto-white epithelium involving the squamo-coulumnar junction, areas of punctation, mosaic pattern or atypical vessels. Unsatisfactory colposcopic examination defined situations where the squamo-columnar junction was not clearly visualized. A tissue biopsy forceps was used to obtain cervical tissue as directed by the abnormal colposcopic area. Participants with normal colposcopic findings were reassured and booked for annual cervical pap cytology. The biopsy specimens were immediately fixed in formalin and transported to the histopathology laboratory for processing and histologic examination and interpretation by a trained pathologist who was blinded to the HR-HPV status of the specimen. The colposcopic findings were illustrated in a diagram and coded for subsequent entry into the study database.

### Procedure for colposcopic examination and histopathology reporting

We used the Leisegang D-10625, Model1DS Ur Nr 55764, Colposcope, Berlin, Germany to examine the cervix. Each participant was placed in lithotomy position and an appropriate size Coscus speculum was introduced into the vagina to expose the cervix for examination. For each participant we first examined the vulva, vagina and the cervix before the application of 3% acetic acid solution. Prior to application of 3% acetic acid, the cervices seen to have excess cervical mucus were washed with 0.9% Normal saline to dislodge excess mucus discharge. Colposcopic abnormalities were described as normal, abnormal or unsatisfactory. Abnormal areas were biopsied using a punch cervical biopsy tissue forceps. The cervical tissues were processed in the histopathology laboratory. The Histopathologist was blinded to the HPV status of the participant and interpreted processed cervical samples either as Normal or CIN 1, CIN 2, CIN 3 or Carcinoma in Situ (CIS) with either well-differentiated, moderate differentiated or poorly differentiated squamous cell carcinoma or other variants if applicable.

### Data collection and statistical analysis

We created a study database that included routinely collected demographic parameters, CD4+ cell count (Partec, Munster, Germany), plasma HIV RNA (viral load; Roche COBAS Amplicor HIV-1 monitor test, version 1.5; Roche Diagnostics, GmbH, Mannheim Germany), HC2 HR-HPV status, date of colposcopic examinations, colposcopic findings and histology results (for those with colposcopic cervical tissue biopsy). To conduct statistical analyses, the database variables were coded for relevant statistical analysis on STATA. All analyses were performed using STATA version 11.0, College station, Texas, USA.

Summary statistics were generated using two-way tables of association to compare baseline socio-demographic characteristics of study participants with normal versus abnormal colposcopic examination findings. The Pearson’s chi square and/or Fisher’s exact test was performed where applicable. We also created relevant indicator variables in order to run univariabe logistic regression analyses to determine odds ratio of having colposcopic abnormalities. In the multivariable logistic regression model the association between positive HR-HPV and abnormal colposcopic findings was adjusted for age category ≥30 years. Because of the relatively small numbers of participants in this pilot data, we estimated the proportions of histologic findings of the cervical biopsies in the study sample. All statistical tests were reported with corresponding 95% confidence interval and p-values. Probability estimates <0.05 were considered statistically significant.

### Human subject protection

Institutional Review Board approval was obtained from the Harvard School of Public Health for identification and enrollment of HIV-infected women into care, treatment and other support services at APIN, JUTH. Secondary use of data approval was also granted by the Harvard School of Public Health to use CD4+ T cell count, viral load and other relevant patient data. The protocol for this study was approved by the JUTH Human Subject Ethics Committee.

## Results

We studied 78 women out of 89 eligible participants (86.5%) who completed colposcopic examination (Table 1). Of the 78 HIV-infected women included in this study, 30 (38.5%) were HR-HPV positive and 48 (61.5%) were HR-HPV negative.

**Table 1:** **Socio-demographic characteristics of HIV infected women with normal cervical cytology who had colposcopic follow up examination in Jos Nigeria**

N = 78			
Variable	Abnormal findings	Normal finding	p-value
Age (years)	34.7 ± 5.5	32.9 ± 4.4	0.069 (t-test)
Parity (N = 78)			
0	2(18.2)	9(81.8)	0.578 (Fisher)
≥1	10(14.9)	57(85.1)	
HR_HPV status (N = 78)			
Positive	8(26.7)	22(73.3)	0.05 (Fisher)
Negative	4(8.3)	44(91.7)	
History of previous abortions (N = 76)			
Yes	6(18.2)	27(81.8)	0.616 (Pearson)
No	6(14.0)	37(86.0)	
History of contraceptive use (N = 78)			
Never	4(21.1)	15(78.9)	0.224 (Fisher)
Past	5(20.8)	1979.2)	
Current	3(8.6)	32(91.4)	
Age_Category (N = 78)			
≤30	2(7.1)	26(92.9)	0.122 (Fisher)
>30	10(20.0)	40(80.0)	
CD4+ cell count category (N = 78)			
<350/mm^3^	4(25.0)	12(75.0)	0.449 (Fisher)
≥350/mm^3^	9(14.5)	53(85.5)	
Viral load category (N = 78)			
<400 copies/ml	11(19.0)	47(81.0)	0.498 (Fisher)
≥400 copies/ml	2(10.0)	18(90.0)	

Socio-demographic characteristics of the study cohort have been summarized in Table 1. The mean age was 32.4 years (SD ±4.6 years) and median age 32 years (IQR 29–36 years).

### Colposcopic abnormalities and HR-HPV

**Figure 1:**
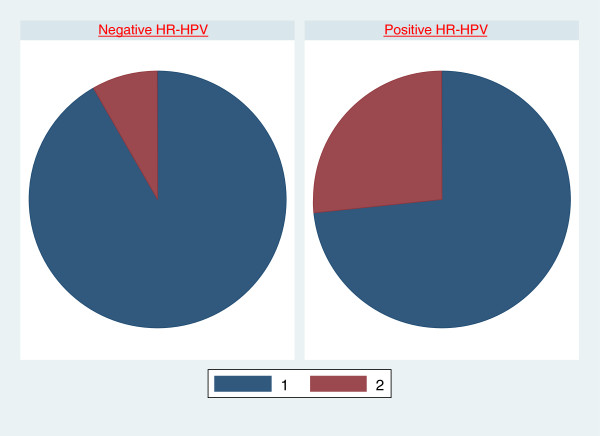
**Normal colposcopic (1) and abnormal colposcopic (2) examinations in HIV infected women with positive HR-HPV compare to those negative for HR-HPV.**

Colposcopic examinations were performed after a mean of 20.1 months from the initial cervical pap cytology and HR-HPV status evaluation. The mean follow up time between initial Pap cytology and subsequent colposcopic evaluation was comparable for women who were positive HR-HPV (20.5 months) and those negative HR-HPV (19.9 months); p-value 0.554. Colposcopy revealed 12 of 78 (15.4%) women with abnormal findings. The odds ratio (OR) of having an abnormal colposcopic examination finding was 4.0 (95% CI: 1.1-14.7) in women who were HR-HPV positive compared to HR-HPV negative; p-value 0.039 (Table 1 and Figure 1).

### Colposcopic abnormalities and histologic diagnoses of CIN/Cancer

Of the twelve women with abnormal colposcopic findings, subsequent histologic examination confirmed CIN 1 in 4 cases (33.3%), CIN 2 in 1 case (8.3%), CIN 3 in 2 cases (16.7%), carcinoma-in-situ (CIS) in 2 cases (16.7%), and normal cervix in 3 cases (25.0%) (Table 2). In all, 5 out 12 (41.7%) of those with abnormal colposcopic findings had a confirmed CIN 2 or greater lesion (CIN2+) of the cervix. Overall, the proportion of women detected with any grade of CIN was 11.5% (9/78) and CIN2+ was 6.4% (5/78) in our cohort. Subsequent analysis of CIN 2+ lesions detected by HR-HPV status showed that 13.3% (4/30) were detected in the HR-HPV positive group compare to 2.1% (1/48) in the HR-HPV negative group (P-value 0.0494).

**Table 2:** **Summary of main outcome for HIV infected women with prior normal cervical cytology with positive HR-HPV or negative HR-HPV in Jos Nigeria**

Outcome	Positive HR-HPV	Negative HR-HPV	P-value
Mean follow up time (Months)	20.5 ± 4.1	19.9 ± 4.3	0.554*
Colposcopic finding			
Abnormal	9(29.0)	22(71.0)	0.001^†^
Normal	3(6.4)	44(93.6)	
Cervical biopsy taken			
Yes	10(83.3)	2(16.7)	0.001^†^
No	21(32.8)	43(67.2)	
Histologic diagnosis			
Normal cervix	1(33.3)	2(66.7)	
CIN I	3(75.0)	1(25.0)	
CIN II	0(0.0)	1(100.0)	
CIN III	2(100.0)	0(0.0)	
Cervical Carcinoma in situ (CIS)	2(100.0)	0(0.0)	
Proportion of CIN 2+ detected	0.133	0.021	0.0494^‡^

*Student t-test for means, ^†^Fisher’s exact p-values, ^‡^t-test of proportion.

### Colposcopic abnormalities, age category and HR-HPV status

In a multivariabe logistic regression model including age ≥ 30 years and HR-HPV positive, we found an OR = 4.9 (95% CI: 0.91-26.2, p value 0.065) for age ≥30 years and an OR = 5.4 (95% CI: 1.4-21.5, p value 0.016) for an HR-HPV positive woman of having an abnormal colposcopic examination finding (Table 3).

**Table 3:** **Univariate and multivariate logistic regression of abnormal colposcopic examination findings in HIV infected women with normal cervical cytology in Jos Nigeria**

Variable	Odds ratio (OR)	95% CI	P–Value
CD4 < 350 cells/mm^3^	1.03	0.30-3.48	0.968
Positive HR-HPV (HC2)	4.00	1.08-14.75	0.037
Previous abortions	0.73	0.21-2.51	0.617
Age ≥30 years	3.58	0.73-17.45	0.115
Viral load >400 RNA copies/ml	0.45	0.10-2.22	0.33
Positive HR-HPV (HC2)	5.44 (AOR)	1.37-21.50	0.016
Age ≥30 years	4.87 (AOR)	0.91-26.17	0.065

AOR = Adjusted Odds Ratio of having an abnormal colposcopic examination findings in a model of positive HR-HPV and Age ≥30 years as co-variates (Hosmer-Lemeshow goodness-of-fit p-value 0.782).

## Discussion

In this pilot study, after a mean follow up time of 20.1 months, we documented abnormal colposcopic findings in 12 of 78 (15.4%) HIV-infected women with prior NCC. Women with HR-HPV had a five-fold higher likelihood of an abnormal colposcopic description compare to those without after controlling for age. We also observed that women with positive HR-HPV had a higher proportion of CIN 2+ cervical abnormalities compare to women negative for HR-HPV. Overall, the proportion of any CIN lesion was 11.5% (9/78) and CIN2+ was 6.4% (5/78) in our cohort.

In a cohort study reported from India [16], the authors described a prevalence of 10.0% of HR-HPV types 16 and 18 in women with cytologically negative Pap smear and any CIN lesion was found in 15% (19 out of 123 women with negative pap cytology) following colposcopy and directed biopsies. A meta-analysis of over 20 studies showed a high prevalence of 36.3% of any HR-HPV among HIV infected women with normal cervical cytology [19] and HIV infected women with high-grades lesions were significantly more likely to be infected with HPV types 11, 18, 33, 51, 52, 53, 58 and 61. Although, the method of HR-HPV detection in our cohort was based on HC2 which shows positivity for any of the following HR-HPV types (16, 18, 31, 33, 35, 39, 45, 51, 52, 56, 58, 59 or 68), the proportion of any CIN lesion detected in our pilot data is comparable with the findings described in India [16]. The India study used specific PCR primers to detect the most common HR-HPV types 16 and 18, which have been reported to account for 70% of cervical cancer globally [8]. Other studies have found a similarly high association of HIV and HR-HPV infection, but reported a higher prevalence and association of higher grade squamous lesions with PCR HR-HPV sub-types 52 and 58 [20]. A recent study on the burden and distribution of HR-HPV types among HIV-infected women in Western Nigeria found a comparably higher prevalence of HR-HPV among HIV-infected women than negative controls with types 16, 35, 58 and 31 being the most prevalent types [21]. Additionally, the ATHENA trial [15], where over 1,500 women with atypical squamous cells of undetermined significance (ASCUS) were examined colposcopically and their specific HR-HPV determined by Cobas 4800, found higher absolute risk of detection of CIN 2+ in women with HR-HPV 16/18 compared to the detection of pooled HR-HPV positive or HR-HPV negative women (24.4%, 14.0% and 0.8% respectively). These findings suggest that HR-HPV testing could increase the detection rate of SILs/Cervical cancer if colposcopic examinations are performed on patients with the most prevalent sub-types causing cancer in a particular setting.

Our pilot data showed that women who had a positive HR-HPV had a five-fold higher likelihood of an abnormal colposcopic examination after adjusting for age. Other factors such as CD4+ T cell count, HIV-1 viral load, previous abortions, and use of contraceptives were not significantly associated with having an abnormal colposcopic examination finding. A prior study in Jos during an era when accessibility and use of antiretroviral drugs was limited [6], found a high prevalence of cervical dysplasia among HIV-infected women whose CD4+ T cell count was less than 200 cell/mm^3^, high viral load (101,781 copies/ml) and those with a clinical evidence of HPV infection on visual inspection. A study from a Brazilian HIV-infected cohort, found a higher association of current cigarette smoking, CD4+ T cell count less than 350 cells/mm^3^, and HR-HPV with colposcopic/histopathologic diagnosis of CIN2+ lesions [22]. Studies have shown a significant association of cervical cancer risk with cigarette smoking, oral contraceptive use, multiparity, impaired cellular immunity and chronic inflammation [23]. Our small study population undergoing successful antiretroviral therapy, may further explain the lack of difference in colposcopic abnormalities with CD4+ T cell count, viral load and other socio-demographic characteristics.

Subsequent colposcopically directed biopsy and histologic diagnosis a significant difference in the proportion of CIN 2+ lesions detected in HIV infected women who were HR-HPV positive compare to those HR-HPV negative. These findings have implications in the practice of cervical cancer screening in our setting. First, since colposcopy and directed biopsy with histologic examination is the gold-standard for the diagnosis of CIN/or invasive cancer; women with HR-HPV are more likely to have such colposcopic examination and directed biopsies thereby increasing the chances of diagnosing high-grade lesions and/or cancer. Results of HPV genotype distribution from meta- analysis data have shown that the HPV genotype distribution in low grades cervical lesions differs from that in cervical cancer [24], suggesting the need to conduct studies on the type-specific HPV genotypes causing cervical cancer in different geographic settings. Several previous studies on the role of colposcopiy in diagnosing squamous intraepithelial lesions and cervical cancer have reported a higher risk of detecting SILs/cancer in women who had prior HR-HPV during follow up compared to HR-HPV negative controls [22, 25, 26].

One alarming finding in our pilot study is the detection of 2 cases of CIS of the cervix following colposcopic examination and directed biopsies in women with prior NCC after only about 20 months of follow up. Indeed, studies have shown an increased hazard of rapid progression from CIN to ICC stages in women infected with HIV compared to HIV-uninfected controls [5]. This finding suggests that women infected with HIV in our setting may benefit from early colposcopic examination of the cervix even when Pap smear cytology is normal.

The major strength of this study lies in the fact that HR-HPV testing is not yet part of our screening for cervical cancer in Nigeria and this is first study examining its possible role in detecting high-grade cervical lesions particularly in HIV-infected women with NCC. We were also able to enroll 86% of the initial cohort and conducted colposcopic examinations irrespective of HR-HPV status. We must however, acknowledge the limitations of a relatively small sample size, which may have reduced the statistical power to control for potential confounding effects on detection of CIN2+ in our cohort. We also did not perform colposcopic examination at the time of initial Pap or HR-HPV determination and the abnormalities/CIN lesions detected at follow up colposcopy examinations may have existed at the time of initial Pap cytology examination.

In all we detected 6.4% (5/78) CIN2 or greater in this small pilot cohort out of which 2 cases were CIS. Since HIV-infected women have a significant hazard of progression from CIN to ICC [5], our data suggests that in situations where facilities for colposcopic examination are limited, efforts should be focused on conducting early colposcopic examination of HIV-infected women with NCC who are over 30 years and positive HR-HPV. We need to study a larger cohort with a longer follow up time, across different regions in Nigeria including HIV positive and negative women to have better insights on the optimal approach for managing HIV infected women with NCC in our setting.
